# Relationship Between Computed Tomography Imaging Features and Clinical Characteristics, Masaoka–Koga Stages, and World Health Organization Histological Classifications of Thymoma

**DOI:** 10.3389/fonc.2019.01041

**Published:** 2019-10-11

**Authors:** Xiaowei Han, Wenwen Gao, Yue Chen, Lei Du, Jianghui Duan, Hongwei Yu, Runcai Guo, Lu Zhang, Guolin Ma

**Affiliations:** ^1^Department of Radiology, China-Japan Friendship Hospital, Beijing, China; ^2^Graduate School of Peking Union Medical College, Chinese Academy of Medical Sciences and Peking Union Medical College, Beijing, China; ^3^Department of Science and Education, Shangluo Central Hospital, Shangluo, China

**Keywords:** computed tomography, thymoma, Masaoka–Koga stage, WHO histological classification, myasthenia gravis

## Abstract

**Objectives:** Computed tomography (CT) is an important technique for evaluating the condition and prognosis of patients with thymomas, and it provides guidance regarding treatment strategies. However, the correlation between CT imaging features, described using standard report terms, and clinical characteristics, Masaoka–Koga stages, and World Health Organization (WHO) classifications of patients with thymomas has not been described in detail nor has risk factor analysis been conducted.

**Methods:** Overall, 159 patients with thymomas who underwent preoperative contrast-enhanced CT between September 2011 and December 2018 were retrospectively reviewed. We assessed the clinical information, CT imaging features, and pathological findings for each patient. A total of 89 patients were specially used to evaluate postoperative recurrence or metastasis between September 2011 and December 2015 to obtain an appropriate observation period. The relationship between CT imaging features and clinical characteristics, Masaoka–Koga stage, and WHO histological classification were analyzed, and related risk factors based on CT imaging features were identified.

**Results:** CT imaging features did not significantly differ based on sex or age. Some imaging features demonstrated significant differences between the groups with and without related clinical characteristics. Contour (odds ratio [OR] = 3.711, *P* = 0.005), abutment ≥50% (OR = 4.277, *P* = 0.02), and adjacent lung abnormalities (OR = 3.916 *P* = 0.031) were independent risk factors for relapse or metastasis. Among all imaging features, there were significant differences between stage I/II and III/IV lesions in tumor size, calcification, infiltration of surrounding fat, vascular invasion, pleural nodules, elevated hemidiaphragm, and pulmonary nodules. Tumor size (odds ratio = 1.261, *P* = 0.014), vascular invasion (OR = 2.526, *P* = 0.023), pleural nodules (OR = 2.22, *P* = 0.048), and pulmonary nodules (OR = 3.106, *P* = 0.006) were identified as independent risk factors. Tumor size, contour, internal density, infiltration of surrounding fat, and pleural effusion significantly differed between low- and high-risk thymomas. Tumor size (OR = 1.183, *P* = 0.048), contour (OR = 2.288, *P* = 0.003), internal density (OR = 2.192, *P* = 0.024), and infiltration of surrounding fat (OR = 2.811 *P* = 0.005) were independent risk factors.

**Conclusions:** Some CT imaging features demonstrated significant correlations with clinical characteristics, Masaoka–Koga clinical stages, and WHO histological classifications in patients with thymomas. Familiarity with CT features identified as independent risk factors for these related clinical characteristics can facilitate preoperative evaluation and treatment management for the patients with thymoma.

## Introduction

Thymomas are thymic epithelial neoplasms most often located in the anterior mediastinum; they account for <1% of all adult malignancies (1, 2). Thymomas are usually slow-growing tumors with local extension and a propensity to spread along the pleura and pericardium, abutting adjacent vessels. Extrathoracic metastases are uncommon (3). Often, these tumors are incidental findings upon physical examination or when patients present with chest pain and shortness of breath. Sometimes thymomas are detected while investigating symptoms associated with paraneoplastic syndrome or autoimmune disorder, such as myasthenia gravis (4, 5). Computed tomography (CT) plays a major role in identifying and characterizing thymomas (6, 7). In the most patients, CT is performed to investigate tumor-related symptoms, identify the tumor clinical stage, and reveal the extent of pathological changes (8–10). Therefore, CT is an important technique for evaluating the condition and prognosis of patients with thymomas, and it provides guidance regarding treatment strategies.

Some previous studies (8, 11–20) focused solely on the relationship between a single CT finding and clinical features, such as myasthenia gravis, adjacent lung invasion, surgical resectability, and postoperative recurrence, whereas others focused on clinical staging or World Health Organization (WHO) histological classification (21–24). One retrospective analysis aimed to identify metastasis or recurrence of thymomas according to longitudinal imaging studies and cross-sectional follow-up imaging after surgery (25). Another study reported that the histological features of aggressive behavior in thymomas were significantly correlated with a decreased doubling time and rapid growth by retrospectively and dynamically evaluating the doubling time (26). Zhao et al. (7) reported the relationship between various CT imaging features and clinical characteristics of thymoma. However, most studies did not systematically investigate the association between CT findings and clinical characteristics, Masaoka–Koga stages, and WHO histological classifications, and no analysis has been conducted to identify risk factors in detail based on CT imaging features. These factors should be further explored and evaluated to provide imaging indicators for the clinical management of thymomas.

This retrospectively study aimed to assess the correlation between CT imaging features, described using standard report terms, and clinical characteristics, Masaoka–Koga stages, and WHO classifications in patients with thymomas. Furthermore, their corresponding risk factors were comprehensively identified using multiple logistic regression analysis.

## Materials and Methods

### Patients

This retrospective study was approved by the Institutional Ethics Review Committee of the China-Japan Friendship Hospital. The patients were identified from a digital medical records database located in the Department of Thoracic Surgery and Pathology. We included patients seen between September 2011 and December 2018 who had preoperative contrast-enhanced CT scans within 2 weeks prior to surgery, and for whom imaging findings were available for re-evaluation. These patients had undergone surgical thymoma resection and the results of subsequent pathological examinations were available for all patients. Of 187 patients, 28 were excluded because of preoperative neoadjuvant chemotherapy or other treatments, in which 17 were receiving steroid therapy because of myasthenia gravis confirmed previously at the first CT examination in our hospital. In total, 159 eligible patients were included, and their clinical information on symptoms, myasthenia gravis, and postoperative recurrence or metastasis was retrospectively obtained from the medical records database. The patients were divided into two subgroup (>49 and ≤ 49 years) according to their average age in order to analyze the relationship between CT imaging features and age by categorical variable.

### CT Imaging and Interpretation

All the patients underwent preoperative cross-section spiral CT examinations. The CT images were obtained with a variety of scanners, including a 16-row multi-detector CT (MDCT) (Toshiba Aquilion, Shipu, Tokyo, Japan), 320-row MDCT (Toshiba Aquilion TM ONE, Shipu, Tokyo, Japan), and a 256-row MDCT (GE revolution, Boston, Massachusetts, USA). All images were taken when the patients were in the supine position with suspended inspiration. Intravenously administered contrast medium was used for all the patients. The images were reconstructed for both the mediastinum (window width, 300–400 HU; window level, 30–50 HU) and lungs (window width, 1,000–1,500 HU; window level, −600~−700 HU). The images with 5-mm slice thicknesses after reconstruction were evaluated, and the original images with 0.6–1.25 mm slice thicknesses were available, if needed, in all cases.

All the CT images were retrospectively reviewed on a picture archiving and communications system by two radiologists who are experts in oncologic and thoracic imaging. The experts were blinded to the patients' clinical details and pathological findings at the time of image interpretations. In case of disagreement, a third radiologist with 23 years of experience specializing in chest tumors addressed the differences and provided the final assessment. The image interpretation criteria used standard reporting terms defined by the International Thymic Malignancy Interest Group (ITMIG) for anterior mediastinal masses suspected to be thymoma (27). The evaluated CT imaging features included the following data on the primary mass and its surrounding structures: lesion location (where tumors in the anterior mediastinum were classified into centrally located, right-sided, and left-sided lesions, and any tumors that were located around or on the line running through the sternum were considered to be centrally located); size in the x, y, and z axis; contour (smooth, single lobulated, and irregular multi-lobulated); internal density (homogenous or heterogeneous); calcifications (without calcification, single or multiple calcifications); infiltration of surrounding fat; tumor abutting of an adjacent mediastinal structure (≥50% or <50%); and direct vascular endoluminal invasion. The following information on surrounding structures were also included: adjacent lung abnormalities, pleural effusion (without, unilateral or bilateral), pleural nodule or nodules, mediastinal lymph node enlargement (>1 cm in short axis on an axial image), phrenic nerve involvement (consistent with elevated hemidiaphragm evaluated on the coronal reconstruction images), and number of pulmonary nodules.

### Clinical Characteristics and Pathologic Evaluation

In this study, clinical characteristics evaluated were clinical symptoms, presence of myasthenia gravis, and recurrence or metastasis. Clinical symptoms included persistent dry cough, shortness of breath, chest pain, and fatigue among others (28). Presence of myasthenia gravis was first diagnosed during hospitalization, without hormone therapy and before CT examination. Patients were followed up by the outpatient department every 3 months for the first year, every 6 months for the second year, and yearly thereafter. Chest CT was performed at 6-month intervals for the first 2 years and at 1-year intervals from the third year. A total of 89 patients who completed follow-up were selected for this study to evaluate the relationship between CT features and postoperative recurrence or metastasis in all 108 patients between September 2011 and December 2015 in order to obtain an appropriate observation period (29).

A compound identification strategy based on pathological findings or results of follow-up was employed for pleural and pulmonary nodule confirmation (27, 30). The postoperative pathologic analysis was performed according to standard procedures (31, 32). The resected mass specimens usually included the adjacent abnormal pleura and lung, and pleural and pulmonary nodular from around the tumor could be identified by gross and microscopic pathological examination. For the distant nodules, most of which showed hard nodules with high density, some cases were confirmed by puncture biopsy and the rest were evaluated by follow-up of at least 1-year.

### Masaoka–Koga Stage and WHO Histological Classification

The final pathological staging was identified according to the modified Masaoka–Koga staging system after a review of the surgical records and pathological reports (33–36). Masaoka–Koga stage III and IV lesions were considered more aggressive than stage I and II lesions. Thymomas were classified according to the 2015 revised WHO histological classification (37, 38), based on the morphology of epithelial cells and the ratio of lymphocytes to epithelial cells. WHO types B2 and B3 were considered to be more malignant than types A, AB, and B1. The invasive and non-invasive groups were divided according to stage, III and IV or I and II, and the high-risk and low-risk groups were divided according to type, B2, and B3 or types A, AB, and B1. When a tumor showed multiple histological components, it was classified based on the predominant component (39).

### Statistical Analysis

We used the an independent two-sample *t*-test for comparison of tumor size with clinical features, stages and histology classification parameters. The χ^2^ test or Fisher's exact tests were employed for comparisons of two or three of the imaging feature classification variables with clinical characteristics, Masaoka–Koga stage I/II and III/IV lesions, and low-risk and high-risk thymomas. Age was converted into categorical variables of >49 or ≤ 49 years. *P* < 0.05 represented statistical significance. Binary logistic regression analysis was used to evaluate CT imaging features as risk factors for the clinical presence of symptoms, myasthenia gravis, postoperative recurrence or metastasis, Masaoka–Koga stage, and WHO histology classifications (13). To identify factors for inclusion in the final model, forward stepping selection was used and the significance level was set at *P* = 0.05. Odds ratio (OR) and 95% confidence intervals (CI) were calculated to demonstrate the association strength of risk factors. The frequency of each imaging feature was quantitatively calculated to intuitive reflect the relative importance of these imaging feature. It is obtained by dividing the case number of one CT feature by the number of patients in the subgroup. All demographic, clinical, and imaging data were analyzed using the SPSS software package (version 23.0, SPSS, Inc., Chicago, IL, USA) and STATA (version 15.0, StataCorp, Texas, USA).

## Results

### Clinical Features and Pathologic Evaluation of Patients

Among 159 patients, 72 (45.3%) were men. Age ranged from 23 to 73 years, with a mean age of 49.82 [±14.53] years. The average tumor size was 6.59 [±2.15] cm, which was reassessed according to the maximum transverse diameter on the axis image. Seventy-five (47.2%) patients had clinical symptoms and 31 (19.5%) had been diagnosed with myasthenia gravis. Of the 159 thymomas, 108 (67.9%) and 51 (32.1%) were categorized as Masaoka–Koga stage I or II and stage III of IV lesions, respectively. The numbers of thymomas according to each WHO type were as follows: 16 (10.1%) type A; 47 (29.5%) type AB; 16 (10.1%) type B1; 45 (28.3%) type B2; 35 (22.0%) type B3. Twenty-five (15.7%) patients had postoperative recurrences or metastases. The median follow-up period after the initial surgical treatment was 35 months (Table 1). Of the sample of 89 patients who completed follow-up, 45 (50.6%) were men. Age ranged from 28 to 73 years, with a mean age of 50.78 [±13.7] years. The average tumor size was 6.83 [±2.4] cm, which was reassessed according to the maximum transverse diameter on the axis image. Forty (44.9%) patients had clinical symptoms and 15 (16.9%) had been diagnosed with myasthenia gravis. Of the 89 thymomas, 60 (67.4%) and 28 (32.6%) were categorized as Masaoka–Koga stage I or II and stage III of IV lesions, respectively. The numbers of thymomas according to each WHO type were as follows: 11 (12.4%) type A; 28 (31.5%) type AB; 14 (15.7%) type B1; 20 (22.4%) type B2; 16 (18.0%) type B3. Twenty-five (20.2%) patients had postoperative recurrence or metastases. The median follow-up period after the initial surgical treatment was 62.9 months (Table 1).

**Table 1:** Clinical characteristics analysis of patients.

**Variables**	**Number of patients (*n* = 158)**	Number of patients completed follow-up (*n* = 89)^*^
Sex		
Male	72 (45.3%)	45 (50.6%)
Female	87 (54.7%)	44 (49.4%)
Age (years)	49.82 ± 14.53	50.78 ± 13.7
Tumor size (cm)	6.59 ± 2.15	6.83 ± 2.4
Clinical symptom		
No	84 (52.8%)	49 (55.1%)
Yes	75 (47.2%)	40 (44.9%)
Myasthenia gravis		
No	128 (80.5%)	74 (83.1%)
Yes	31 (19.5%)	15 (16.9%)
Masaoka-Koga		
I	57 (35.8%)	31 (34.8%)
II	51 (32.1%)	29 (32.6%)
III	38 (23.9%)	23 (25.9%)
IV	13 (8.2%)	6 (6.7%)
WHO classification		
A	16 (10.1%)	11 (12.4%)
AB	47 (29.5%)	28 (31.5%)
B1	16 (10.1%)	14 (15.7%)
B2	45 (28.3%)	20 (22.4%)
B3	35 (22%)	16 (18%)
Postoperative relapse or metastasis		
No	–	71 (79.8%)
Yes	–	18 (20.2)
Median observation period (months)	–	62.9

* *Relapse or metastasis based on a total of 89 patients with the median observation period of 62.9 months*.

In 53 cases with pleura nodule or nodules on CT imaging, 28 cases were confirmed by postoperative pathological examination, of which six were metastatic nodules adhered to adjacent pleura separated from tumor. The remaining cases were confirmed malignant or benign based upon growth or stabilization after at least 12 months follow-up, of which two cases were distant metastases nodules. Of 45 cased with pulmonary nodule or nodules, 18 were pathologically confirmed by examination of postoperative specimens or puncture biopsies, of which five were metastatic nodules in the adjacent or distant pulmonary parenchyma. The remaining cases were considered to be benign or malignant according after at least 15 months of follow-up, of which no cases were considered metastasis nodules. A total of 12 patients had both pleural and pulmonary nodules observed on CT images.

### Relationship Between CT Imaging Features and Clinical Characteristics

**Figure 1:**
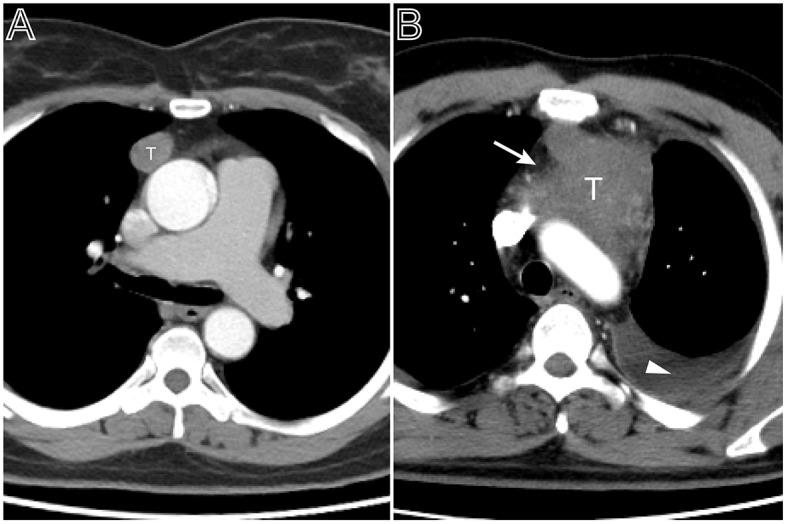
Tumor size and pleural effusion are associated with patient's symptoms. **(A)** Axial contrast-enhanced chest CT image obtained at the level of the aortic arch showed a small thymoma with a smooth contour (Masaoka–Koga stage I and WHO classification A), which was first found at the time of physical examination in a 49-year-old woman. **(B)** A 53-year-old man with chief complains of cough, chest pain symptoms. Axial contrast-enhanced chest CT image obtained at the level of below the aortic arch showed a larger thymoma with lobulated contour and infiltration of surrounding fat (arrows), accompanied with left pleural effusion (Masaoka–Koga stage III and WHO classification B2).

The CT imaging features did not significantly differ based on sex or age. Tumor size (*P* ≤ 0.001), pleural effusion (*P* = 0.012), and elevated hemidiaphragm (*P* = 0.026) did not significantly differ between those with and without symptoms (Figure 1). There were significant differences between the group with myasthenia gravis and the group without myasthenia gravis in terms of tumor size (*P* = 0.001), infiltration of surrounding fat (*P* = 0.01), pleural nodules (*P* = 0.005), elevated hemidiaphragm (*P* = 0.008), and pulmonary nodules (*P* = 0.006). The tumor contours (*P* = 0.001), calcification (*P* = 0.029), tumor abutment of ≥50% (*P* = 0.006), and adjacent lung abnormalities (*P* = 0.039) between the groups with and without postoperative recurrence or metastasis also significantly differed. Among the imaging features, the tumor location, internal density, abutment <50%, vascular invasion, and lymph node enlargement did not significantly differ based on the clinical characteristics (Table 2).

**Table 2:** Analysis of the relationship between CT imaging features and clinical characteristics.

**CT imaging features**	**Sex**		**Age**		**Symptom**		**MG**		**Relapse or MT**	
	**M/F**	***P*-value**	**>49/≤49**	***P*-value**	**No/Yes**	***P*-value**	**No/Yes**	***P*-value**	**No/Yes**	***P*-value**
Location										
Predominant middle	25/22		24/23		(26/21)		42/5		13/2	
Predominant left	26/34	0.407	29/31	0.672	(31/29)	0.92	48/12	0.123	38/9	0.313
Predominant right	21/31		23/29		(27/25)		38/14		20/7	
Tumor size/*P*-value										
6.59 ± 2.15	6.27/6.84	0.101	6.70/6.49	0.546	5.64/7.66	<0.001^*^	6.32/7.71	0.001^*^	6.45/6.94	0.393
Contours/*P*-value										
Smooth	30/37		31/36		37/30		56/11		31/2	
Single-lobulated	36/38	0.501	39/35	0.516	41/33	0.213	59/15	0.544	33/9	0.001^*^
Multi-lobulated	6/12		7/11		6/12		13/5		7/7	
Internal density/*P*-value										
Homogenous	37/43	0.805	44/36	0.095	41/39	0.688	64/16	0.872	35/7	0.427
Heterogeneous	35/44		33/46		43/36		64/15		36/11	
Calcification										
No	45/52		43/54		51/46		82/15		50/8	
Single calcification	17/24	0.845	20/21	0.178	44/32	0.502	33/8	0.06	15/6	0.029
Multiple calcification	10/11		14/7		9/12		13/8		6/4	
Infiltration of fat/*P*-value										
No	42/52	0.854	49/45	0.633	53/41	0.28	82/12	0.010^*^	39/7	0.224
Yes	30/35		30/35		31/34		46/19		32/11	
Abutment ≥ 50%										
No	38/56	0.139	48/46	0.424	50/44	0.913	76/18	0.894	52/7	0.006^*^
Yes	34/31		29/36		34/31		52/13		19/11	
Abutment <50%										
No	31/49	0.096	37/43	0.58	41/39	0.472	63/17	0.574	34/9	0.873
Yes	41/38		40/39		44/35		65/14		37/9	
Vascular invasion										
No	53/57	0.271	54/56	0.802	62/48	0.181	(93/17	0.054	47/8	0.09
Yes	19/30		23/26		22/27		35/14)		24/10	
Adjacent lung abnormalities										
No	40/42	0.361	37/45	0.389	43/39	0.919	70/12	0.116	50/8	0.039^*^
Yes	32/45		40/37		41/36		58/19		21/10	
Pleural effusion										
No	38/44		43/39		52/30		67/14		34/9	
Unilateral	26/31	0.696	24/33	0.612	27/30	0.012^*^	42/15	0.343	29/8	0.654
Bilateral	8/12		10/10		5/15		18/2		8/1	
Pleural nodule										
No	52/54	0.091	57/49	0.056	61/45	0.092	92/14	0.005^*^	46/9	0.249
Yes	19/34		20/33		23/30		36/17		25/9	
Lymph node enlargement										
No	57/69	0.982	61/65	0.994	64/62	0.315	103/23	0.441	54/14	0.878
Yes	15/18		16/17		20/13		25/8		17/4	
Elevated hemidiaphragm										
No	54/63	0.713	58/59	0.63	68/49	0.026^*^	100/17	0.008^*^	48/13	0.706
Yes	18/24		19/23		16/26		28/14		23/5	
Pulmonary nodule										
No	58/56	0.054	53/61	0.437	63/51	0.328	98/16	0.006^*^	48/11	0.603
Yes	14/31		24/21		21/24		30/15		23/7	

*MG, Myasthenia gravis*.

*Relapse or MT: relapse or metastasis based on a total of 89 patients with the median observation period of 62.9 months*.

* *Significant differences*.

### Relationship Between CT Imaging Features and Masaoka–Koga Stage and WHO Classification

**Figure 2:**
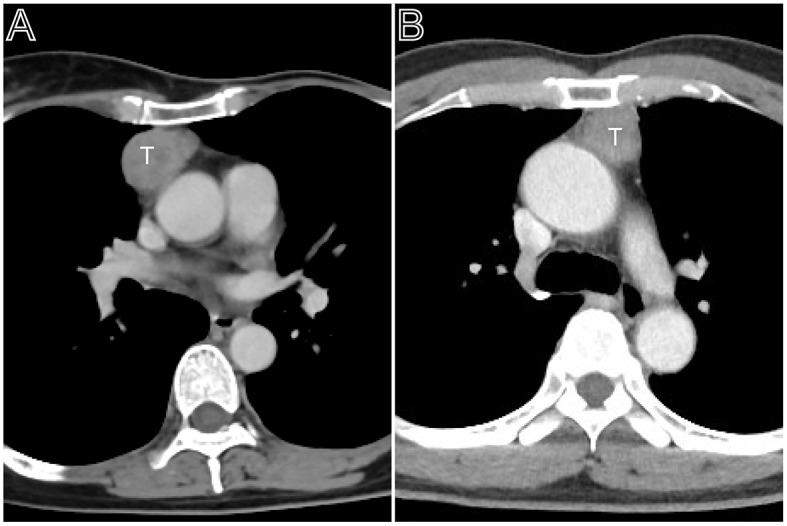
Masaoka–Koga stage II thymomas. **(A)** Axial contrast-enhanced chest CT image showed a thymoma with a clear contour (Masaoka–Koga stage IIa and WHO classification B1), which was first found at the time of physical examination in a 55-year-old woman. **(B)** A 50-year-old woman mainly complains of general fatigue and subsequently was confirmed myasthenia gravis. Axial contrast-enhanced chest CT image obtained at the level of the left pulmonary trunk showed a thymoma with a slight lobulated contour (Masaoka–Koga stage IIb and WHO classification AB).

**Figure 3:**
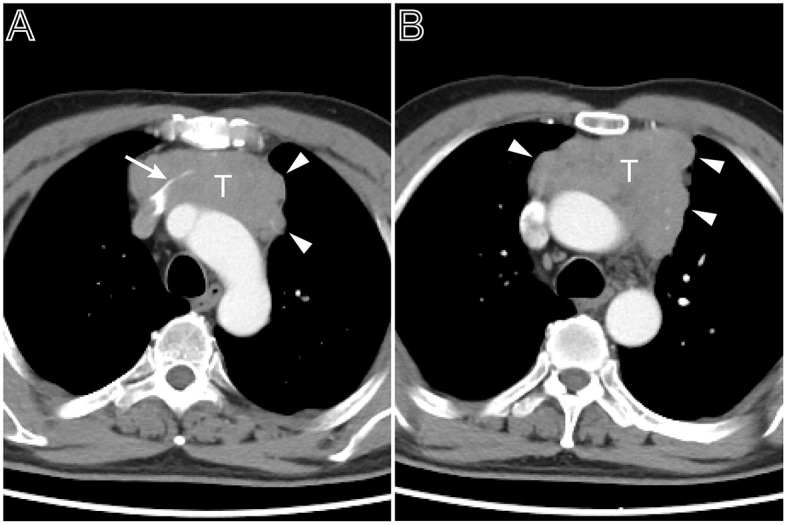
A multi-lobulated heterogeneous thymoma with vascular invasion. Axial contrast-enhanced chest CT image obtained at the level of the aortic arch demonstrated a thymoma in a 54-year-old man complained of bosom frowsty and chest pain (Masaoka–Koga stage III and WHO classification B1). The lesion directly invaded the left brachiocephalic vein (**A**, white arrow). The tumor showed the heterogeneous attenuation distributions and multi-lobulated contour (**A**,**B**, arrow head), the aortic arch was pushed backward.

**Figure 4:**
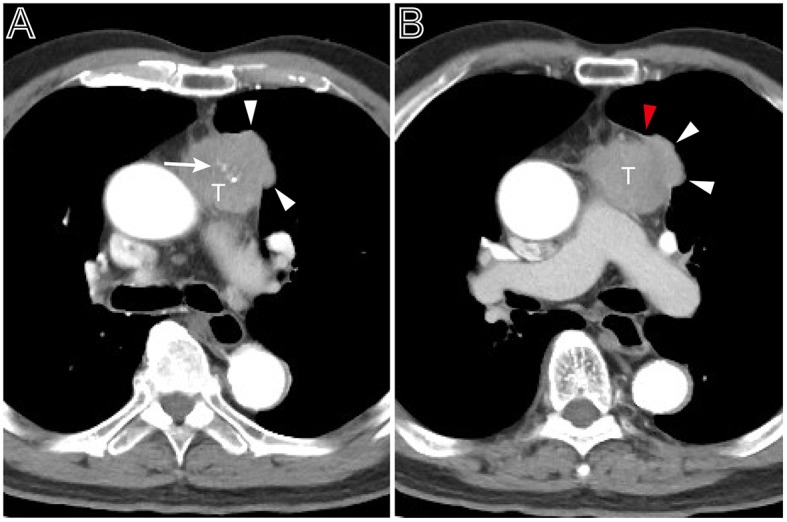
A heterogeneous thymoma accompanied with pleural nodule. **(A)** Axial contrast-enhanced chest CT image obtained at the level of the pulmonary trunk demonstrated a multi-lobulated thymoma with internal multiple calcification lesions (white arrow) in a 69-year-old man with myasthenia gravis (Masaoka–Koga stage IVa and WHO classification B3). **(B)** Showed the adjacent thickened pleura (red arrow head) and pleural nodule (white arrow head).

**Figure 5:**
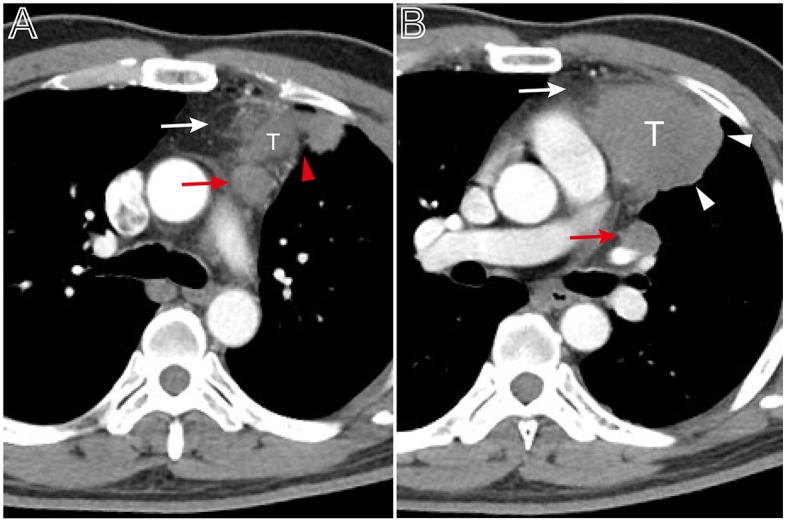
A thymoma with infiltration of surrounding fat and lymph node enlargement. Axial contrast-enhanced chest CT image obtained at the level of the pulmonary trunk demonstrated a thymoma in a 49-year-old man (Masaoka–Koga stage IVb and WHO classification B3). **(A,B)** Showed the thickened pleura (red arrow head), infiltration of surrounding fat (white arrows), and bulging margin (white arrow head). The lymph node enlargement appeared at the superior level of the lesion (**A**, red arrow) and at the level of left hilum (**B**, red arrow).

When compared to the stage I/II lesions, the stage III/IV thymomas were larger, had more irregular contours, exhibited infiltration of the surrounding fat, calcification, and vascular invasion based on the morphology (Figures 2–4). Among all imaging features, there were statistically significant differences between stage I/II and III/IV lesions in tumor size (*P* = 0.001), calcification (*P* = 0.041), infiltration of surrounding fat (*P* = 0.031), vascular invasion (*P* = 0.015), pleural nodules (*P* = 0.024), elevated hemidiaphragm (*P* = 0.022), and pulmonary nodules (*P* = 0.001) (Table 3). Among all imaging features, tumor size (*P* = 0.029), contours (*P* = 0.011), internal density (*P* = 0.021), infiltration of surrounding fat (*P* = 0.016), and pleural effusion (*P* = 0.046) significantly differed between the low- and high-risk thymomas (Table 4). Tumors classified as B2 and B3 tended to exhibit a larger size, irregular contours, infiltration of surrounding fat, internal heterogeneous density, and pleural effusion based on the morphology appearance in this study (Figures 1, 4, 5).

**Table 3:** Analysis of the relationship between CT imaging features and the subgroups of Masaoka–Koga stage.

**CT imaging features**	**Masaoka–Koga (I/II)**	**Masaoka–Koga (III/IV)**	***P*-value**
Location			0.849
Predominant middle	30	17	
Predominant left	40	20	
Predominant right	36	16	
Tumor size			0.001^*^
6.59 ± 2.15	6.21 ± 2.11	7.35 ± 2.04	
Contours			0.867
Smooth	45	22	
Single-lobulated	50	24	
Multi-lobulated	11	7	
Internal density			0.823
Homogenous	54	26	
Heterogeneous	52	27	
Calcification			0.041^*^
No	67	30	
Single calcification	30	11	
Multiple calcification	9	12	
Infiltration of fat			0.031^*^
No	69	25	
Yes	37	28	
Abutment ≥ 50%			0.068
No	68	26	
Yes	38	27	
Abutment <50%			0.911
No	53	27	
Yes	53	26	
Vascular invasion			0.015^*^
No	80	30	
Yes	26	23	
Adjacent lung abnormalities			0.432
No	57	25	
Yes	49	28	
Pleural effusion			0.392
No	55	26	
Unilateral	36	21	
Bilateral	15	6	
Pleural nodule			0.024^*^
No	77	29	
Yes	29	24	
Lymph node enlargement			0.678
No	85	41	
Yes	21	12	
Elevated hemidiaphragm			0.022^*^
No	84	33	
Yes	22	20	
Pulmonary nodule			0.001^*^
No	85	29	
Yes	21	24	

* *Significant differences*.

**Table 4:** Analysis of the relationship between CT imaging features and the subtypes of WHO histological classification.

**CT imaging features**	**Low-Risk(A/AB/B1)**	**Hight-Risk(B2/B3)**	***P*-value**
Location			0.237
Predominant middle	27	20	
Predominant left	29	31	
Predominant right	21	31	
Tumor size			0.029^*^
6.59 ± 2.15	6.21 ± 1.85	6.95 ± 2.36	
Contours			0.011^*^
Smooth	38	29	
Single-lobulated	36	38	
Multi-lobulated	3	15	
Internal density			0.021^*^
Homogenous	46	34	
Heterogeneous	31	48	
Calcification			0.375
No	50	47	
Single calcification	16	25	
Multiple calcification	11	10	
Infiltration of fat			0.016^*^
No	53	41	
Yes	24	41	
Abutment ≥ 50%			0.623
No	44	50	
Yes	33	32	
Abutment <50%			0.814
No	38	42	
Yes	39	40	
Vascular invasion			0.105
No	58	52	
Yes	19	30	
Adjacent lung abnormalities			0.093
No	45	37	
Yes	32	45	
Pleural effusion			0.046^*^
No	47	34	
Unilateral	20	37	
Bilateral	11	10	
Pleural nodule			0.369
No	54	52	
Yes	23	30	
Lymph node enlargement			0.051
No	66	60	
Yes	11	22	
Elevated hemidiaphragm			0.055
No	62	55	
Yes	15	27	
Pulmonary nodule			0.528
No	57	57	
Yes	20	25	

* *Significant differences*.

### Screening for Risk Factors Based on Binary Logistic Regression

**Figure 6:**
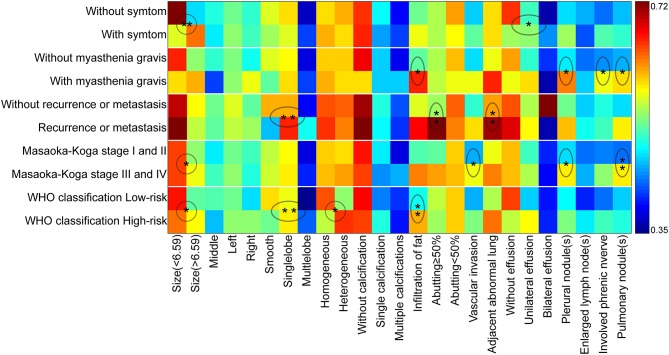
The schematic diagram of CT imaging features as risk factors for clinical characteristics, Masaoka–Koga stage, and WHO histological classification. The X-axis displayed the CT imaging features and Y-axis indicated the clinical characteristics, Masaoka–Koga stage, and WHO histological classification. The ratio of occurrence frequency of one imaging feature was quantitatively calculated in one subgroup and size of ratio value was represented by color bar (With and without relapse or metastasis were based on a total of 89 patients). The circle and oval circle showed the imaging features as risk factors for the related clinical characteristics, Masaoka–Koga stage, and WHO histological classification. The single star (^*^) indicated the significant difference (*P* < 0.05) and the double stars (^**^) indicated the significant difference (*P* < 0.01) by the binary logistic regression analysis.

Binary logistic regression analysis indicated that tumor size (OR = 1.687, *P* < 0.001) and pleural effusion (OR = 1.743, *P* = 0.033) were risk factors for the patients' symptoms. The infiltration of surrounding fat (OR = 2.873, *P* = 0.022), pleural nodules (OR = 2.734, *P* = 0.028,), elevated hemidiaphragm (OR = 2.975, *P* = 0.021), and pulmonary nodules (OR = 2.708, *P* = 0.033,) were identified as risk factors for patients with myasthenia gravis. Contour (OR = 3.711, *P* = 0.005), abutment ≥50% (OR = 4.277, *P* = 0.02), and adjacent lung abnormalities (OR = 3.916, *P* = 0.031) were risk factors for postoperative recurrence or metastasis. Tumor size (OR = 1.261, *P* = 0.014), vascular invasion (OR = 0.526, *P* = 0.023), pleural nodules (OR = 2.22, *P* = 0.048), and pulmonary nodules (OR = 3.106, *P* = 0.006) were identified as risk factors for Masaoka–Koga stage I/II and III/IV lesions. Tumor size (OR = 1.183, *P* = 0.048), Contour (OR = 2.288, *P* = 0.003), internal density (OR = 2.192, *P* = 0.024), and infiltration of surrounding fat (OR = 2.811, *P* = 0.005) were risk factors for low- and high-risk thymomas according to the WHO classification (Table 5). The frequency of each imaging features were quantitatively calculated and intuitively illustrated alongside risk factors by heat map (Figure 6).

**Table 5:** Screening of risk factors for clinical characteristics, Masaoka-Koga stage and WHO classification by binary logistic regression analysis.

**Clinical characteristics, stage and classification**	**CT imaging features**	***P*-value**	**OR (95% C.I.)**
Presence of symptom	Tumor size	<0.001	1.687 (1.373–2.127)
	Pleural effusion	0.033	1.743 (1.046–2.906)
Presence of Myasthenia gravis	Infiltration of fat	0.022	2.873 (1.166–7.080)
	Pleural nodule	0.028	2.734 (1.114–6.710)
	Elevated hemidiaphragm	0.021	2.975 (1.177–7.518)
	Pulmonary nodule	0.033	2.708 (1.081–6.784)
Postoperative relapse or metastasis^*^	Contour	0.005	3.711 (1.491–9.241)
	Abutment ≥ 50%	0.020	4.277 (1.255–14.572)
	Adjacent lung abnormalities	0.031	3.916 (1.133–13.533)
Masaoka-Koga stage	Tumor size	0.014	1.261 (1.048–1.516)
	Vascular invasion	0.023	2.526 (1.138–5.604)
	Pleural nodule	0.048	2.220 (1.008–4.488)
	Pulmonary nodule	0.006	3.106 (1.392–6.931)
WHO classification	Tumor size	0.048	1.183 (1.002–1.398)
	Contour	0.003	2.288 (1.323–3.957)
	Internal density	0.024	2.192 (1.120–4.322)
	Infiltration of fat	0.005	2.811 (1.375–5.746)

* *Postoperative relapse or metastasis: based on a total of 89 patients with the median observation period of 62.9 months*.

*OR, Odds ratio; 95% C.I., 95% confidence interval*.

## Discussion

All patients underwent preoperative cross-section spiral CT examinations with same body position and methods; however, the CT images were obtained with three different types of scanners in this study. Uniform axial 5-mm images were reconstructed for the evaluation of CT features from both mediastinum and lung windows, with the same ranges of window width and level, and the original images with 0.6–1.25 mm slice thicknesses were available ensuring the consistency of this evaluation. Although it has been previously reported (40) that MG has no effect on the survival rate of patients with thymoma, patients with a history of hormonal therapy were excluded from our study. A total of 89 patients with a median follow-up period 62.9 months were specially used to evaluate the relationship between CT features and postoperative recurrence or metastasis on the basis of their long observation period (29), although similar studies allowed a minimum follow-up time of 6 months (25). Pleural and pulmonary nodules were diagnosed with a compound identification strategy based on either pathology results or follow-up examination because it is difficult to confirm all nodules by pathology alone, even in patients with other malignancies (30). For the distant nodules, most of which were hardened nodules with high density, this may be due to the fact that thymoma with distant metastasis are rare and these nodular lesions usually shaped in a history of chronic inflammation (28). Elevated hemidiaphragm was evaluated by coronal images reconstructed from original images with 0.6–1.25 mm slice thicknesses instead by section observation. Because the supine position is not ideal for evaluating the position of diaphragm, the elevated hemidiaphragm findings may be overestimated in some cases. However, the suspended inspiration and bilateral contrast observations can partially offset this error. In addition, there were no low-density cystic thymomas in this study. Some cases of cystic and necrotic changes in tumors were described as either heterogeneous-density according to terms recommended by ITMIG (27).

In this study, those ≤ 49 and >49 years of age did not have any significant differences in imaging features. Zhao et al. (7) reported that the infiltration of the surrounding fat was associated with age. However, most studies did not report a relationship between CT imaging features and age (41–44). Tumor size, pleural effusion, and elevated hemidiaphragm significantly differed between the groups with or without symptoms; however, tumor size and pleural effusion were independent risk factors. Most studies did not focus on the relationship between CT imaging features and symptoms, with the exception of one study which identified negative findings (7). In our study, tumor size, infiltration of surrounding fat, pleural nodules, elevated hemidiaphragm, and pulmonary nodules significantly differed between patients with and without myasthenia gravis. The infiltration of surrounding fat, pleural nodules, elevated hemidiaphragm, and pulmonary nodules were identified as independent risk factors for patients with myasthenia gravis. These results were similar to those of previous studies (7, 21, 45–48). Klimiec et al. (16) suggested that routine CT imaging can effectively identify thymomas; however, radiologists should be cautious in interpreting these images when differentiating between thymic hyperplasia and a normal thymus in patients with myasthenia gravis, as they are not reliable. This study found that contour, abutment ≥50%, and adjacent lung abnormalities were risk factors for postoperative recurrence or metastasis upon logistic regression analysis. These findings are somewhat similar to previous results (7, 18, 21, 49, 50). However, some studies reported that tumor size or histological subgroups were related to postoperative recurrence or metastasis (19, 25, 26). Kato et al. suggested that the maximum tumor diameter and the contact length between the tumor contour and the lung was significantly associated with pleural recurrence after complete resection of the thymoma (13). These differences may be a result of the thymoma staging and the different focus of the authors. The features of tumor location, internal density, abutment <50%, vascular invasion, and lymph node enlargement did not significantly differ when the clinical features were analyzed. These results were consistent with those from most of the previous studies (13, 18, 19, 26, 41). Therefore, we speculate that these features do not play a significant role in the clinical features of patients with thymoma (51, 52).

Previous studies (7, 16, 21, 23, 26, 41) on thymoma CT were based on the Masaoka–Koga clinical staging system. In the present study, tumor size, calcification, infiltration of surrounding fat, vascular invasion, pleural nodules, elevated hemidiaphragm, and pulmonary nodules significantly differed between stage I/II and III/IV lesions. Tumor size, vascular invasion, pleural nodules, and pulmonary nodules were identified as independent risk factors in multiple regression analysis. Zhao et al. reported that the tumor contour, calcification, infiltration of surrounding fat, and degree of abutment of vessels were related to the Masaoka–Koga stage subtypes (7). Ozawa et al. considered that larger tumor size and necrosis was related to Masaoka–Koga stage III/IV (21). Choe et al. reported that the histological features of aggressive tumor behavior were significantly correlated with decreased doubling time and rapid growth (26). These results fully demonstrated that thymoma is a type of heterogeneous tumor, and its clinical staging is associated with multiple imaging features. However, the tumor samples collected in each study may differ. Stage III/IV thymomas were larger, had more irregular contours, and exhibited infiltration of surrounding fat, calcification, and vascular invasion compared with stage I/II lesions based on morphology (13, 26, 41, 51).

The tumor size, contours, internal density, infiltration of surrounding fat, and pleural effusion significantly differed between low-risk and high-risk thymomas among all imaging features in our study. However, upon logistic regression analysis, pleural effusion was not an independent risk factor. These results were similar to those of previous studies (7, 21, 53), although CT could not more effectively differentiate the WHO histological subtypes of thymomas (7, 54). Johnson et al. studied the pathologic response of tumors to neoadjuvant treatment and found an association between CT of tumor response after neoadjuvant treatment and pathologic response (51). CT imaging was of limited value in discriminating between thymoma subtypes. Tumor size of ≥7 cm was considered a B3 thymoma in a previous study (20), but this finding was not evident in our study.

Our study demonstrated that the CT findings were associated with clinical characteristics, Masaoka–Koga stage, and WHO histological classification of thymomas, in which tumor size, contour, internal density, abutment ≥50%, fat infiltration, vascular invasion, adjacent lung abnormalities, pleural effusion, pleural and pulmonary nodules, and elevated hemidiaphragm were identified as independent risk factors. To the best of our knowledge, there have been no prior studies on detailed risk factors evaluation based on various imaging features (24, 55–57). Based on the logistic regression analysis, the elevated hemidiaphragm were not independent risk factors for the patients' symptoms. Tumor size was not an independent risk factor for thymomas in patients with myasthenia gravis. Calcification, infiltration of surrounding fat, and elevated hemidiaphragm were not independent risk factors for Masaoka–Koga stage. Additionally, pleural effusion was not an independent risk factor for the WHO classification of thymomas. However, these results can provide reference indicators for clinical management and should not be regarded as absolute results based on retrospective imaging data statistics due to confounding factors that are difficult to detect (13, 51, 58, 59).

This study was subject to several limitations. First, the incidence rate of thymoma was very low and data of some variables in this study were retrospectively collected and analyzed. Second, the CT scanners used in this study and their parameters were dissimilar and diaphragm evaluation was based on the coronal reconstruction images. However, we believe that these parameters do not strongly affect our evaluation of the manifestations of thymoma that can be assessed using CT. Finally, the number of eligible cases was slightly smaller especially for the evaluation of recurrence or metastasis. Future studies should endorse multi-center collaborations to include more thymoma cases in order to obtain more accurate conclusions. High-dimensional data from quantitative analysis of volume and radiomics of thymomas should be used for the exploratory and excavated research in order to get more relevant results in the next phase (9, 59–61).

In conclusion, this study demonstrated that part of CT imaging features had significant correlations with clinical characteristics, Masaoka–Koga clinical stage, and WHO histological classification of thymomas. Consequently, familiarity with certain CT features analyzed as independent risk factors can facilitate preoperative evaluation, decision-making and postoperative management for patients with thymomas.

## Data Availability Statement

All datasets generated for this study are included in the manuscript/supplementary files.

## Ethics Statement

Written informed consent was obtained from the individual(s) for the publication of any potentially identifiable images or data included in this article.

## Author Contributions

XH acquired, analyzed and explained data, and drafted the manuscript. WG, LD, and YC analyzed and explained the imaging data. JD, HY, and RG acquired the clinical information and revised the manuscript. LZ and GM designed the study and revised the manuscript.

### Conflict of Interest

The authors declare that the research was conducted in the absence of any commercial or financial relationships that could be construed as a potential conflict of interest.
